# Survival and pleurodesis outcome in patients with malignant pleural effusion – a systematic review

**DOI:** 10.1515/pp-2020-0147

**Published:** 2021-02-08

**Authors:** Maged Hassan, Elinor Harriss, Rachel M. Mercer, Najib M. Rahman

**Affiliations:** Chest Diseases Department, Alexandria Faculty of Medicine, Alexandria, Egypt; Bodleian Health Care Libraries, University of Oxford, Oxford, UK; Oxford Respiratory Trials Unit, University of Oxford, Oxford, UK

**Keywords:** malignancy, pleural effusion, pleurodesis, survival, systematic review

## Abstract

Malignant pleural effusion (MPE) is a common condition that presents with progressive breathlessness. Long term solutions are often required due to recurrence of effusion after simple drainage. Pleurodesis is one of the main options resorted to for long term control of MPE. There is data to suggest there may be a survival benefit for patients with MPE who achieve successful pleurodesis. A systematic review was carried out to explore this correlation and results suggest that there could be a survival difference according to pleurodesis outcome in patients with MPE. Fifteen studies (reported in 13 papers) were included; 13 (86.6%) of the studies showed survival difference in favour of pleurodesis success. The median [interquartile range] difference in survival between the two groups among the different studies was five [3.5–5.8] months. Most of the included studies suffered moderate to severe risk of bias and, thus, large prospective studies of patients undergoing pleurodesis are required to ascertain this effect.

## Introduction

Malignant pleural effusion (MPE) is a common condition complicating the clinical course of about 50% of patients with disseminated malignancy [1]. Patients usually present with progressive breathlessness, and the effusion typically recurs after simple pleural aspiration. Longer term solutions are often resorted to and these involve either performing pleurodesis (i.e. obliterating the pleural space by inducing adhesions) or inserting an indwelling pleural catheter (IPC) for long term drainage [2].

Patients with MPE suffer from limited survival, with median survival between 3 and 12 months [2]. Recent observations suggest that patients with MPE secondary to breast cancer who underwent pleurodesis plus receiving chemotherapy had better survival than those who had chemotherapy alone [3]. Another recent study of patients with MPE due to different primaries who underwent pleurodesis found that survival was longer in patients who achieved successful pleurodesis in comparison to those who experienced fluid recurrence [4].

A systematic review of available literature was carried out to search for studies reporting on survival of patients with MPE who underwent pleurodesis and to inspect whether there was difference in survival according to pleurodesis outcome.

## Methods

**Figure 1:**
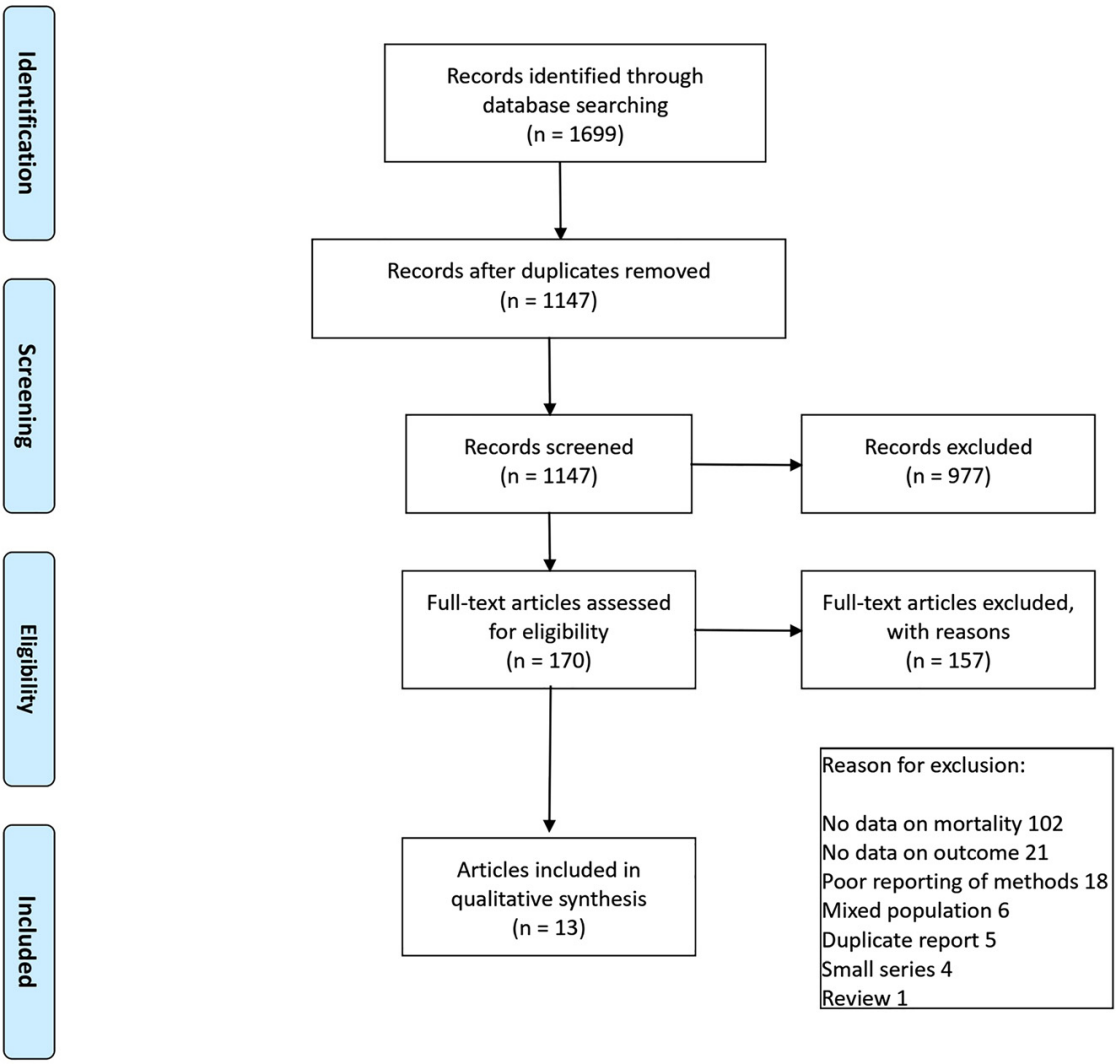
PRISMA flow diagram of the different stages of the systematic review.

The systematic review was carried out in accordance with the Preferred Reporting Items for Systematic Reviews and Meta-Analyses (PRISMA) guidance and the protocol was registered with the Prospero database (ref. CRD42018115874). The primary outcome of the review along with the full search strategy and risk of bias assessment are reported elsewhere [5]. The literature search was carried out on Medline, Embase and Cochrane Database of Clinical trials on 12th November 2018 and updated for the purpose of this review on 1st July 2020. Figure 1 presents the PRISMA flow diagram of the different stages of the review.

All full articles of adult patients with MPE who underwent pleurodesis that provided data on survival were included. Studies were excluded if the method of determining pleurodesis outcome was not documented clearly. Two authors screened the titles and reviewed the retrieved full articles. Any disagreement regarding inclusion/exclusion decisions was resolved by discussion.

Due to the methodological heterogeneity between studies reporting hazard ratios and the statistical difficulty in combining results of studies reporting median survival times, a narrative synthesis of the results rather than a meta-analysis was presented.

## Results

The literature search returned 1,147 titles. Following duplicate screening of the search results against the exclusion criteria, 170 papers were deemed potentially relevant and retrieved in full for review (Figure 1). Thirteen reports providing data on 15 different studies (totalling 2,103 patients) were included [4], [6], [7], [8], [9], [10], [11], [12], [13], [14], [15], [16], [17]; the summary of which is presented in Table 1 and Supplementary Table. Eleven (73.3%) of the included studies were retrospective, three (20%) were prospective observational and a single study (6.7%) was a post-hoc analysis of a clinical trial. Weighted mean age of studied patients was 68.45 (95% CI 67.7 to 69.1) years. The most common primary malignancies were breast cancer, lung cancer and mesothelioma. Thirteen (86.6%) of the 15 studies showed survival difference in favour of pleurodesis success. Eleven of these studies provided the median survival times of the two patient groups; those who achieved pleurodesis and those with pleural fluid recurrence. The median [interquartile range] difference in survival between the two groups among the different studies was five [3.5–5.8] months. The majority of the studies included (14 of 15) showed moderate to high risk of bias.

**Table 1:** Summary of the included studies in the systematic review.

Study	Number	Study design	Primary malignancy	Pleurodesis agent	Percent success	HR of poor survival (95% CI)	Median survival (success vs. failure)	Factors controlled for in multi-variate analysis
Viallat 1996	360	Retrospective	Miscellaneous	Talc	93	UA	7.6 vs. 2.6 months, p=0.001	N/A
Love 2003	60	Retrospective	Miscellaneous	Talc	47.6	UA	346 vs. 133 days, p=0.03	N/A
Kolschmann 2005	85	Retrospective	Miscellaneous	Talc	89.4	UA	No difference in 180 days survival, p=0.44	N/A
Trotter 2005	202	Retrospective	Miscellaneous	Talc	88.1	UA	107 vs. 45 days, p=0.26	N/A
Stefani 2006	109	Prospective	Miscellaneous	Talc	83	UA	9.4, vs. 5.8 m, p=0.048	N/A
AK 2009	42	Retrospective	Mesothelioma	Talc	61.9	2.59 (1.20–5.61)	UA	Chemotherapy
Nikbakhsh 2011	50	Prospective	Miscellaneous	Bleomycin	88	UA	No difference in 180 days survival, p=0.57	N/A
Rena 2015	172	Retrospective	Mesothelioma	Talc	76	2.54 (1.73–4.40)	UA	Mesothelioma subtype, cancer stage, performance status, elevated serum CRP, elevated platelet count
Hsu 2016	26	Prospective	Lung & breast	Minocycline	64	UA	220 vs. 112 days, p=0.015	N/A
Santos 2017	202	Retrospective	Miscellaneous	Talc	70.7	UA	400 vs. 170 days, p=0.01	N/A
Leemans 2018	155	Retrospective	Miscellaneous	Talc	78	1.92 (1.09–3.33)	169 vs. 66 days, p=0.02	N/A
Hsu 2019a	205	Retrospective	Miscellaneous	Minocycline	69	3.70 (2.43–5.55)	414 vs. 100 days, p<0.001	Performance status, extrapleural metastasis
Hsu 2019b	109	Retrospective	Miscellaneous	Minocycline	70.5	2.67 (1.66–4.34)	259 vs. 102 days, p<0.001	Performance status, extrapleural metastasis
Hassan 2019a	266	RCT	Miscellaneous	Talc	78	1.62 (1.09–2.34)	12 vs. 7.3 months, p=0.004	Primary malignancy, unexpandable lung
Hassan 2019b	60	Retrospective	Miscellaneous	Talc	48	2.85 (1.08–7.50)	16 vs. 5 months, p=0.007	Primary malignancy, systemic therapy, LDH level, unexpandable lung

HR, hazard ratio; CI, confidence interval; UA, unavailable; N/A, not applicable; RCT, randomised controlled trial.

## Discussion

The results of this systematic review demonstrate a survival difference according to pleurodesis outcome in patients with MPE. This was shown by several studies of different designs and on patients with different primary malignancies. Despite the consistency in the findings of the included studies regarding the difference in survival, the overall level of evidence presented is not robust enough to make firm conclusions since the majority of the studies included in the systematic review are retrospective/observational in nature with substantial risk of bias.

In patients with MPE several factors affect pleurodesis outcome, survival, or both. In order to ascertain if there is true correlation between pleurodesis outcome and survival, it is crucial to control for possible confounders. Some of the studies reported multi-variate analyses, mostly by performing Cox proportional hazards model, to control for clinically relevant factors. Performance status is one of the most important factors affecting survival in such cohort of patients. Two of the included studies (both retrospective in nature) [11, 15] examined survival in patients with MPE after undergoing pleurodesis, and after controlling for patients’ performance status, and a difference in survival was noticed according to pleurodesis outcome. Another important factor that potentially affects both pleurodesis outcome and survival is the type of malignancy, and this was controlled for in retrospective [4, 11] and prospective studies [4]. Initiation of active oncological treatment potentially affects both survival and pleurodesis outcome, and two studies [4, 9] controlled for this factor. The status of lung expandability has a strong bearing on the success of pleurodesis and can reflect heavier malignant infiltration of the visceral pleural which would impact survival negatively. Lung expandability has been controlled for in the multi-variate analyses in the two studies reported in the paper by Hassan et al. [4] showing positive correlation between pleurodesis success and survival. Additionally, the extent of spread of malignancy is a strong predictor of survival. In patients with primary pleural malignancy (mesothelioma), the extent is defined as the TNM stage which was controlled for by the study by Rena et al. [11]. Patients with MPE due to non-pleural malignancy are all in stage four, but the extent of extra-pleural spread can be a marker of disease burden. The study by Hsu et al. [15] reported that this factor was controlled for in the analysis but did not provide the actual data.

Different mechanisms can explain the association between pleurodesis failure and poorer survival. Theoretically, the mere persistence of pleural fluid can potentially act as a medium for further propagation of malignancy and a barrier for oncological treatment to reach its target. This is supported by the observation that patients who failed pleurodesis and those who were treated with IPC had shorter survival times in comparison to patients who successfully achieved pleurodesis [15]. There is *in vitro* data that show that MPE fluid allows perpetuation of cell lines from primary and secondary pleural malignancies and that the fluid causes the malignant cells to resist the effects of cytotoxic medications [18]. Alternatively, pleural inflammation, which is known to be associated with successful pleurodesis [1] could have a role in the defence against cancer, and thus patients who fail pleurodesis and who mount weaker inflammatory response do not benefit from a potential anticancer effect of inflammation.

This review has limitations which mandate caution in interpreting the results. Besides the retrospective nature of most of the included studies, several important confounders (e.g. institution of oncological therapies) have only been controlled for in such studies which limit the robustness of evidence. None of the included prospective studies had a pre-hoc aim of studying the survival difference in patients with MPE undergoing pleurodesis and therefore, it is not possible to rule out reporting bias.

In conclusion, this systematic review suggests that there could be a survival difference according to pleurodesis outcome in patients with MPE. This signal needs to be further explored in large prospective cohorts of patients undergoing pleurodesis with appropriate methods applied to control for important confounding factors. The results of the SIMPLE trial (ISRCTN16441661) are expected to be published soon which will provide stronger evidence regarding predictors of survival in patients with MPE. Studies are needed to probe possible mechanisms that can explain the correlation between survival and pleurodesis outcome. Such data is needed and if difference is proved to be genuine, this can potentially affect future management of patients by placing stronger recommendation for pleurodesis as opposed to other forms of fluid control in eligible patients.

## References

[1] Mierzejewski M , Korczynski P , Krenke R , Janssen JP . Chemical pleurodesis – a review of mechanisms involved in pleural space obliteration. Respir Res 2019;20:247. 10.1186/s12931-019-1204-x. 31699094 PMC6836467

[2] Bibby AC , Dorn P , Psallidas I , Porcel JM , Janssen J , Froudarakis M , . ERS/EACTS statement on the management of malignant pleural effusions. Eur Respir J 2018;52 1800349 10.1183/13993003.00349-2018. 30054348

[3] Hirata T , Yonemori K , Hirakawa A , Shimizu C , Tamura K , Ando M , . Efficacy of pleurodesis for malignant pleural effusions in breast cancer patients. Eur Respir J 2011;38:1425–30. 10.1183/09031936.00171610. 21565923

[4] Hassan M , Mercer RM , Maskell NA , Asciak R , McCracken DJ , Bedawi EO , . Survival in patients with malignant pleural effusion undergoing talc pleurodesis. Lung Canc 2019;137:14–8. 10.1016/j.lungcan.2019.09.003. 31521977

[5] Hassan M , Gadallah M , Mercer RM , Harriss E , Rahman NM . Predictors of outcome of pleurodesis in patients with malignant pleural effusion: a systematic review and meta-analysis. Expert Rev Respir Med 2020;14:645–54. 10.1080/17476348.2020.1746647. 32213100

[6] Viallat JR , Rey F , Astoul P , Boutin C . Thoracoscopic talc poudrage pleurodesis for malignant effusions. A review of 360 cases. Chest 1996;110:1387–93. 10.1378/chest.110.6.1387. 8989050

[7] Kolschmann S , Ballin A , Gillissen A . Clinical efficacy and safety of thoracoscopic talc pleurodesis in malignant pleural effusions. Chest 2005;128:1431–5. 10.1378/chest.128.3.1431. 16162739

[8] Trotter D , Aly A , Siu L , Knight S . Video-assisted thoracoscopic (VATS) pleurodesis for malignant effusion: an Australian Teaching Hospital’s experience. Heart Lung Circ 2005;14:93–7. 10.1016/j.hlc.2005.02.004. 16352262

[9] Ak G , Metintaş M , Yildirim H , Metintaş S , Dündar E , Erginel S , . Pleurodesis in follow-up and treatment of malignant pleural mesothelioma patients. Tuberk Toraks 2009;57:22–31. 19533434

[10] Nikbakhsh N , Pourhasan Amiri A , Hoseinzadeh D . Bleomycin in the treatment of 50 cases with malignant pleural effusion. Caspian J Intern Med 2011;2:274–8. 24049586 PMC3770504

[11] Rena O , Boldorini R , Papalia E , Mezzapelle R , Baietto G , Roncon A , . Persistent lung expansion after pleural talc poudrage in non-surgically resected malignant pleural mesothelioma. Ann Thorac Surg 2015;99:1177–83. 10.1016/j.athoracsur.2014.11.050. 25669666

[12] Hsu L-H , Hsu P-C , Liao T-L , Feng A-C , Chu N-M , Kao S-H . Pleural fluid osteopontin, vascular endothelial growth factor, and urokinase-type plasminogen activator levels as predictors of pleurodesis outcome and prognosticators in patients with malignant pleural effusion: a prospective cohort study. BMC Canc 2016;16:463. 10.1186/s12885-016-2529-1. PMC4944509 27411914

[13] Stefani A , Natali P , Casali C , Morandi U . Talc poudrage versus talc slurry in the treatment of malignant pleural effusion. Eu J Cardiothorac Surg 2006;30:827–32. 10.1016/j.ejcts.2006.10.002. 17113008

[14] Santos PS , Marques MA , Cruz C , Monteiro H , Fradinho F . Predictors of talc slurry pleurodesis success in patients with malignant pleural effusions. Rev Port Pneumol 2017;23:216–20. 10.1016/j.rppnen.2017.01.008. 28606378

[15] Hsu LH , Feng AC , Soong TC , Ko JS , Chu NM , Lin YF , . Clinical outcomes of chemical pleurodesis using a minocycline. Ther Adv Respir Dis 2019;13 175346661984123 10.1177/1753466619841231. PMC6454655 30945619

[16] Love D , White D , Kiroff G . Thoracoscopic talc pleurodesis for malignant pleural effusion. ANZ J Surg 2003;73:19–22. 12534732 10.1046/j.1445-2197.2003.02616.x

[17] Leemans J , Dooms C , Ninane V , Yserbyt J . Success rate of medical thoracoscopy and talc pleurodesis in malignant pleurisy: a single-centre experience: pleurodesis in malignant pleurisy. Respirology 2018;23:613–7. 10.1111/resp.13252. 29320805

[18] Cheah HM , Lansley SM , Varano della Vergiliana JF , . Malignant pleural fluid from mesothelioma has potent biological activities: biological capabilities of pleural fluid. Respirology 2017;22:192–9. 10.1111/resp.12874. 27560254

