# Supplementary material for: Survival and pleurodesis outcome in patients with malignant pleural effusion – a systematic review
Source: Pleura Peritoneum. 2021 Feb 8;6(1):1–5. doi: 10.1515/pp-2020-0147 (PMC8223802; doi:10.1515/pp-2020-0147)
Supplement: Supplementary file 1 [file pp-06-20200147-s001.docx]

| **Supplementary table: details of the study cohorts and interventions in the systematic review** | | | | |
| --- | --- | --- | --- | --- |
| **Study** | **Most common malignancies** | **Pleurodesis method** | **Chemotherapy** | **Performance status** |
| Viallat 1996 | Breast 33%, Mesothelioma 24%, Lymphoma 10% | Talc poudrage under sedation (2/3) or general anaesthesia (1/3) | no data | no data |
| Love 2003 | Breast 30%, Mesothelioma 23%, Lung 19% | VATS talc poudrage | no data | no data |
| Kolschmann 2005 | Lung 47%, Breast 16%, Mesothelioma 10% | LAT talc poudrage | no data | All ECOG PS <3 |
| Trotter 2005 | Lung 30%, Breast 18%, Mesothelioma 15% | VATS talc poudrage | no data | 83% ASA > or = 3 |
| Stefani 2006 | Lung 45%, Breast 26%, Mesothelioma 9% | VATS talc poudrage 66%, talc slurry 34% | no data | 81% with ECOG PS <3, no effect on pleurodesis outcome |
| AK 2009 | Mesothelioma | LAT talc poudrage or slurry | Chemotherapy and pleurodesis outcome independently correlated with survival | All Karnofsky performance score > or = 60 |
| Nikbakhsh 2011 | Breast 40%, Lung 28% | Bleomycin through chest tube | no data | All Karnofsky performance score > or = 50 |
| Rena 2015 | Mesothelioma | VATS talc poudrage | 91% on chemotherapy, associated with better survival | 82% with ECOG PS <2, with HR of death with higher PS |
| Hsu 2016 | Lung, 52% Breast 31%, Others 17% | Minocycline slurry | no data | no data |
| Santos 2017 | Lung 65%, Breast 15%, Haematological 8% | Talc slurry | no data | no data |
| Leemans 2018 | Lung 45%, Breast 22%, Ovary 10% | LAT talc poudrage | 40% on chemotherapy, no effect on pleurodesis success | no data |
| Hsu 2019a | Lung 48%, Breast 33%, Others 19% | Minocycline slurry | all patients on guideline-based oncological treatment | ECOG PS collected, no data given |
| Hsu 2019b | Lung 66%, Breast 26%, Others 8% | Minocycline slurry | all patients on guideline-based oncological treatment | ECOG PS collected, no data given |
| Hassan 2019a | Mesothelioma 40%, Breast 11%, Lung 19% | LAT talc poudrage 66%, talc slurry 34% | no data | All ECOG PS <3 |
| Hassan 2019b | Mesothelioma 36%, Breast 23%, Lung 22% | LAT talc poudrage 56%, talc slurry 44% | More common in those with pleurodesis success and better survival | no data |

ASA: American society of anaesthesiology, ECOG: European Cooperative Oncology group, PS: performance status, HR: hazard radio, LAT: local anaesthetic thoracoscopy, VATS: video-assisted thoracoscopic surgery
